# Organizational role stress among medical school faculty members in Iran: dealing with role conflict

**DOI:** 10.1186/1472-6920-7-14

**Published:** 2007-05-29

**Authors:** Soleiman Ahmady, Tahereh Changiz, Italo Masiello, Mats Brommels

**Affiliations:** 1 Department of Learning, Informatics, Management, and Ethics, Karolinska Institutet, KI, 171 76 Stockholm, Sweden, and National Public Health Management Center, Tabriz University of Medical sciences, IR-Iran; 2 Medical Education Research Centre, Isfahan University of Medical Sciences, IR-Iran; 3 Department of Learning, Informatics, Management, and Ethics, Karolinska Institutet, Sweden; 4 Medical Management Centre, Department of Learning, Informatics, Management, and Ethics, Karolinska Institutet, Sweden, and Department of Public Health, University of Helsinki, Finland

## Abstract

**Background:**

Little research has been conducted to investigate role stress experienced by faculty members in medical schools in developing countries. This becomes even more important when the process of reform in medical education has already taken place, such as the case of Iran. The objectives of this study were to investigate and assess the level and source of role-related stress as well as dimensions of conflict among the faculty members of Iranian medical schools. Variables like the length of academic work, academic rank, employment position, and the departments of affiliation were also taken into consideration in order to determine potentially related factors.

**Methods:**

A survey was conducted at three different ranks of public medical schools. The validated Organizational Role Stress Scale was used to investigate the level of role stress and dimensions of role conflict among medical faculty members. The response rate was 66.5%.

**Results:**

The findings show that role stress was experienced in high level among almost all faculty members. All three studied medical schools with different ranks are threatened with relatively the same levels of role stress. Specific differences were found among faculty members from different disciplines, and academic ranks. Also having permanent position and the length of services had significant correlation with the level of role stress. The major role- related stress and forms of conflict among faculty members were role overload, role expectation conflict, inter-role distance, resource inadequacy, role stagnation, and role isolation.

**Conclusion:**

The most role-related stressors and forms of conflict among faculty members include too many tasks and everyday work load; conflicting demands from colleagues and superiors; incompatible demands from their different personal and organizational roles; inadequate resources for appropriate performance; insufficient competency to meet the demands of their role; inadequate autonomy to make decision on different tasks; and a feeling of underutilization. The findings of this study can assist administrators and policy makers to provide an attractive working climate in order to decrease side effects and consequences of role stress and to increase productivity of faculty members. Furthermore, understanding this situation can help to develop coping strategies in order to reduce role-related stress.

## Background

A movement towards greater accountability and transparency has occurred in higher education recently and consideration of faculty issues is a direct response. Today, every university administrator is aware of the challenges that higher education faces [1]. Some of these are typical challenges as managerial and financial, while others are new, such as expanded participation and distance education [2]. One of the most challenging aspects for current higher education institutes is the proliferation of roles that faculty members have to undertake [3], during their everyday educational and clinical practice. Medical knowledge is increasing exponentially, the disease patterns are changing, the approach to health-care delivery and medical education is shifting, and also professional roles and boundaries are being modified [4].

Over the past three decades, significant steps have been taken to improve the quality of health care delivery and medical education in Iran. Initially, there were few comprehensive universities in Iran, which included medical schools as well as art, science, and technical schools. The limited health workforce who graduated from Iranian universities did not meet community needs. Many partially qualified foreign professionals were employed by the government to fulfill these needs. The problem of quality in health care and medical education was the next concern which resulted in a very large organizational change by implicating all managerial and provisional levels of medical education and health services. One major step was the gradual integration of medical education into health services with the purpose of health empowerment and a more relevant training for the workforce [5,6]. The integration sought also to provide a better response to the health needs of the community while broadening learning/teaching and research activities. However, there is concern that following the process of reform in the health service and medical education in Iran, faculty members are facing major challenges, which causes stress on them. So, the aim of this study is to investigate and assess levels, and sources of role stress.

Medical education in Iran has been now completely integrated into the healthcare system [5-8]. However, this has resulted in a complex organizational change. A single "Ministry of Health and Medical Education" was formed and became the responsible organization for both medical education and health services at the national level. Correspondingly, universities of Medical Sciences and Health Services are responsible bodies at the provincial or territory level. As a result of this reorganization, it may be expected that the new system exposes faculty to further and challenging roles. Medical university faculty members are now expected to teach, deliver community services, conduct research, and perform managerial tasks.

Yet little is known, and not much research has reported about role problems of faculty in medical universities in Iran [9,10], although role problems and specifically "role stress" may adversely affect personal and organizational behavior and performance [11,12].

### Organizational stress

As organizations become more complex, the potentiality for stress increases. Stress is a consequence of socio-economic complexity and to some extent is a stimulant as well. Therefore, one should find ways of using stress productively, and reduce dysfunctional stress. The term "stress" will be used here to refer to such terms and concepts as strain and pressure. The concept of role and its two related aspects, role space and role set, have a built-in potential for conflict and stress [13].

### Role space

Role Space has three main variables: self, the role under question, and the other roles one occupies. Any conflict among these is referred to as role space conflict. These conflicts may take several forms as Inter-Role Distance, Self/Role Conflict, Role-Expectation Conflict, Personal Inadequacy, and Role Stagnation [13,14].

### Role set

Role set is the role system within the organization of which roles are part and by which individual roles are defined. Role set conflicts take the forms of Role Ambiguity, Role Overload, Role Erosion, Resource Inadequacy, and Role Isolation. The above dimensions of conflict are worth considering in relation to organizational role stress [13,14].

### Brief definition of each dimension

Inter-Role Distance: conflict between one's organizational role and other roles, e.g., between travel on the job and spending time with one's family.

Role Stagnation: a feeling of stagnation and lack of growth in the job because of few opportunities for learning and growth.

Role-Expectation Conflict: conflicting demands placed on one from others in the organization, e.g., producing excellent work but finishing under sever time restraints. Personal Inadequacy: lack of knowledge, skill, or preparation to be effective in a particular role.

Self/Role Conflict: a conflict between one's personal values or interests and one's job requirements. Role Ambiguity: unclear feedback from others about one's responsibilities and performance.

Role Overload: too much to do and too many responsibilities to do everything well.

Role Erosion: a decrease in one's level of responsibility or a feeling of not being fully utilized.

Resource Inadequacy: lack of resources or information necessary to perform well in a role.

Role Isolation: feelings of being isolated from channels of information and not being part of what is happening.

### Consequences of role stress: the current environment

Researchers investigating organizational stress have noted a number of dysfunctional outcomes resulting from stress, both physiological and psychological, which ultimately affect the functioning and effectiveness of the organization and its employees [15]. Thus, the impact of work stressors has been assessed not only in terms of its effect on the organization, decreased productivity, turnover and decreased job satisfaction, but also in terms of the emotional impact on the workers [16]. Furthermore, "burnout" is usually a consequence of long-term involvement in emotionally demanding situations and ineffective coping with long-term stress [17].

Stress and burnout are important issues for health care professionals because they are considered as significant risks to health and well-being of physicians, and are also associated with reduced quality of health care, attrition, and reduced commitment to practice [18].

Stress among university faculty has received considerable attention in past decades [19-21]. Harden [22], in a study on stress, pressure and burnout in teachers, wrote:

"Alarming statements have been issued in the education literature about the growing prevalence of teacher stress and burnout and the adverse effect this has on the learning environment and on the achievement of the educational goals". He also mentioned that in a conflict of roles medical teachers are confronted by demands and expectations from a number of sources, and those cannot all be met within a given time. Fisher [23] noted that university staffs are expected to teach, meet tutorial, laboratory or seminar commitments and at the same time carry out research, run experiments, obtain research funds, and write papers and books.

Levels of stress and depression in teachers and instructors have been shown to be high or higher than those in doctors and dentists [24]. Many of the stressors are similar: workload, lack of resources, poor relationships with colleagues, and unrealistic expectations from managers.

It is obvious that university faculty members are not exempt from problems associated with role stress and burnout [25]. A study of organizational role stress in relation to job burnout among university teachers has been conducted in 2001 in India [15]. The results of the study indicated that organizational role stress is highly correlated with job burnout among all ranks of faculty members, and sources of stress included excessively high self-expectations, the pressure to secure financial support for research, insufficient time to keep up with developments in the field, inadequate salary, manuscript preparation, role overload, conflicting job demands, slow progress on career advancement, frequent interruptions, and long meetings [19,26]. Klenke-Hamel et al [27], in a study that included university faculty, revealed the relationship between role strains, tension, job satisfaction, and the propensity to leave the job. Faculty who were experiencing more stress than they could cope with were more likely to withdraw from student-professor interactions, be less accessible to students, and be less involved in the departmental decision making and committee work.

### Specific aims of the study

As mentioned earlier, there is an ongoing concern about the faculty members in Iranian medical schools. There are arguments that following the process of reform in the health services and medical education in Iran brings major challenges to faculty members, who are expected to undertake various roles like teaching, research, community services and administration in the broad territory under the control of their university.

Although the consequences of role stress, role conflicts, and burnout among other professionals and in other settings are relatively well documented [24,28-31], understanding the causes of role stress among Iranian medical schools faculty is of paramount importance for their well-being and the delivery of health care and medical education.

Therefore, the objectives of the present study are to investigate the level and the source of job-related stress among Iranian medical schools faculty members. Variables such as experience (length of academic work), academic rank, employment position, and the department of affiliation are also taken into consideration in order to determine potentially related factors.

## Methods

### Design and setting

In order to accomplish the above set of research objectives, a survey was conducted at three different types of medical schools. This was a convenience sampling among all Iranian medical schools and was based on their national ranking and good access to samples. The study had been ethically approved by the Iranian national ethical committee, Ministry of Health and Medical Education (reference number:P/391). The informed consent was obtained from all participants.

Classification of medical universities in three medical schools was based on the number of faculty members, number of students, conducting or not conducting postgraduate educational programs, carrying out residency or subspecialty programs, and resource allocation. The three resulting medical schools were: type I had the largest number of faculty members and students and they conducted most undergraduate and postgraduates educational medical programs; type II had all of the above mentioned indices but at lower extents; and type III normally had very few undergraduate educational medical programs and no postgraduate ones in their system.

Regarding the departments of affiliation, as according to the diversity of departments the roles of faculty are different, faculty members were divided into three main categories consisting of medical basic sciences, medical clinical, and surgical clinical departments.

The three medical schools were Isfahan as type I (large), Urmia as type II (middle), and Lorestan as type III (small). The samples contained permanent, probationary and non permanent faculty with the rank of professor, associate professor, assistant professor, and instructor. Using census method, contact information of 525 faculty members (345 from Isfahan, 105 from Urmia, and 74 from Lorestan medical schools) was obtained and faculty members on leave or sabbatical were not considered.

### Instrument for data gathering

The Organizational Role Stress Scale (ORSS) [13] was used to measure individuals' role stress and several forms of conflict within an organization. The instrument is relevant for measuring job stress in this study and has confirmed reliability and validity [13,14], even though it has never been tested with Iranian medical faculty. This scale is comprised of the following dimensions:

Inter-Role Distance (IRD), Role Stagnation (RS), Role-Expectation Conflict (REC), Role Erosion (RE), Role Overload (RO), Role Isolation (RIs), Personal Inadequacy (PI), Self/Role conflict (S/RC), Role Ambiguity (RA), and Resource Inadequacy (RIn) [14].

Part one of the questionnaire consisted of demographic items: subjects were asked to identify themselves according to the department at which they work, their rank, length of service, employment position, gender, and age. Part two consisted of ORSS which has 50 statements with five-point Likert scale regarding role stress and anchored by: 'If you never or rarely feel that way', 'If you occasionally feel that way', 'If you sometimes feel that way', 'If you frequently feel that way', and 'If you always feel that way', respectively. The scores for each role stress dimension ranged from a minimum of 0 to a maximum of 20 and total scores ranged from 0 to 200, as the scale has ten dimensions and each dimension has five items. As the instrument was to be translated to Farsi, the translation was done by two independent translators, and re-checked by a back-translator, to be sure about the accuracy of the translation process. Also a pre-test was conducted with several faculty members in Isfahan to test the face validity of the instrument in the Iranian context, and minimal changes then applied.

### Survey implementation

Survey packages were mailed to participants according to the lists of faculty members from the three medical schools. The questionnaire was coded to ensure confidentiality, and responders were asked to send it back to their Educational Development Centers (EDC). To increase the response rate of questionnaires, follow up activities such as telephone reminders and re-sending of the questionnaires were done.

### Data analysis

Data were analyzed using SPSS (Statistical Package for Social Sciences, version 11.5). To identify levels of role stress and differences within the dimensions of role stress among faculty members, data were analyzed using parametric statistics. The results were also compared to the norm values obtained in previous studies with the same instrument [14], to check how they differ. A variety of statistical analyses were applied to the data, including t-test, Pearson's product-moment correlation and Analysis of Variance (ANOVA); Duncan's post hoc was applied when appropriate. To use ANOVA for comparison of data from different departments of affiliation, all of them were classified into three major groups (i.e., basic medical sciences, clinical medical, and clinical surgical), based on having clinical work, and being responsible for operating room procedures. A series of multiple linear regressions was used to measure the relationship between individual and organizational factors. The Stepwise method was used. Also all dimensions of role stress were used in the analysis as control or dependent variables. The threshold for statistical significance was considered as p value < .05 for all statistical analyses.

## Results

The number of returned questionnaires was 349 out of 525 (66.5%), 10 were returned entirely unmarked and 6 without complete information. At last, 333 cases were considered for analysis. The respondents were 261 males (78.4%) and 72 females (21.9%). In terms of length of service at the medical school, 149 (44.7%) of respondents had worked as school faculty for less than 10 years, 134 (40.2%) had been at the school for 10-20 years, 50 (15%) for more than 20 years. Their age range was between 29 to 64 years.

Regarding the academic rank of faculty members there were 19 Professors (5.7%), 53 Associate Professors (15.9%), 231 Assistant Professors (69.4%) and 30 Instructors (9%).

The employment status of 251 respondents was permanent (75.4%), 56 probationary (16.8%), meaning a transitional status to permanent, and 26 temporary (7.8%), that is academic services for a given period of 4–8 years.

The mean score for total role stress in all medical schools was 73 (for medical school type I was 73.4; medical school type II was 72.1; and medical school type III was 72.5) while the norm of total mean score was reported as 51 [14]. The mean scores for all 10 dimensions of ORSS in the three medical schools as well as reported norms are presented in table 1.

**Table 1:** A comparison of role stress scores among medical schools.

**Role Stress Dimensions**	Isfahan Medical School	Urmia Medical School	Lorestan Medical School	Total	Norm score	Statistical Inference
Total Stress Score	73.5 SD ± 32.6	71.7 SD ± 29.1	72.5 SD ± 25.4	73.0 SD ± 30.8	Mean = 51 Low = 25 Median = 45 High = 82	High
Inter-Role Distance	8.7 SD ± 5.0	8.4 SD ± 4.6	10.2 SD ± 4.4	8.9 SD ± 4.8	Mean = 5 Low = 2 Median = 5 High = 8	High
Role Stagnation	8.0 SD ± 4.8	8.0 SD ± 4.5	7.6 SD ± 3.4	7.9 SD ± 4.5	Mean = 5 Low = 2 Median = 5 High = 8	High
Role-Expectation Conflict	8.8 SD ± 4.3	9.0 SD ± 4.3	7.6 SD ± 3.9	8.6 SD ± 4.3	Mean = 4.3 Low = 2 Median = 4 High = 7	High
Role Erosion	7.8 SD ± 4.2	7.5 SD ± 3.2	8.3 SD ± 3.5	7.8 SD ± 3.9	Mean = 9.3 Low = 7 Median = 9 High = 12	Low
Role Overload	6.9 SD ± 4.5	7.1 SD ± 4.7	7.0 SD ± 3.9	7.0 SD ± 4.5	Mean = 3.3 Low = 1 Median = 3 High = 6	High
Role Isolation	9.3 SD ± 4.4	8.6 SD ± 4.0	8.9 SD ± 3.9	9.1 SD ± 4.3	Mean = 6 Low = 3 Median = 6 High = 9	High
Personal Inadequacy	5.6 SD ± 3.7	5.4 SD ± 3.5	4.6 SD ± 3.6	5.4 SD ± 3.7	Mean = 4.6 Low = 2 Median = 4 High = 8	Median
Self/Role Conflict	4.9 SD ± 4.0	5.0 SD ± 3.9	4.6 SD ± 3.2	4.8 SD ± 3.9	Mean = 5.6 Low = 3 Median = 5 High = 9	Median
Role Ambiguity	5.5 SD ± 4.6	5.0 SD ± 3.5	4.7 SD ± 3.8	5.2 SD ± 4.2	Mean = 3.6 Low = 1 Median = 3 High = 7	Median
Resource Inadequacy	7.8 SD ± 4.1	8.3 SD ± 3.7	8.7 SD ± 4.0	8.1 SD ± 4.0	Mean = 5 Low = 2 Median = 5 High = 8	High

According to the one-way ANOVA, there were not significant differences in the means of ORSS dimensions and grand total scores among the three studied medical schools (Table 2). Regarding faculty members affiliated to the three groups of departments, the mean score for total role stress in three groups of departments were 73.6 for surgical clinical, 73.5 for medical clinical, and 63.5 for medical basic sciences. The stress levels within faculty's department of affiliation were compared using ANOVA (table 2). Duncan's post hoc showed that mean scores of IRD, RS, REC, RO, S/RC, RA and grand total in medical basics sciences faculty members were significantly different from those in medical clinical and surgical departments. Basic sciences faculty members to some extent showed the lowest scores on all dimensions of role conflicts in comparison to the other groups.

**Table 2:** Summary of One-way ANOVA on dimensions of role stress and selected variables.

Role Stress Dimension	Med. School			Department 3group			Academic rank			Employ. position		
				
	Mean	F	p	Mean	F	p	Mean	F	P	Mean	F	p
IRD	8.7	2.2	NS*	8.9	7.9	<.001	8.9	2.4	.06	8.9	4.1	.01
RS	7.9	.15	NS	7.9	3.39	.03	7.9	4.3	.005	7.9	1.3	NS
REC	8.6	1.8	NS	8.6	4.9	.008	8.6	1.9	NS	8.6	2.6	NS
RE	7.8	.69	NS	7.8	3.2	NS	7.8	1.2	NS	7.8	2.7	NS
RO	7.0	.09	NS	7.0	6.5	.002	7.0	1.9	NS	7.0	2.4	NS
RIs	9.1	.8	NS	9.1	1.9	NS	9.1	4.0	.008	9.1	.30	NS
PI	5.4	1.5	NS	5.4	2.2	NS	5.4	4.7	.003	5.4	.56	NS
SRC	4.8	.21	NS	4.8	4.1	.01	4.8	4.8	.003	4.8	1.4	NS
RA	5.2	.85	NS	5.2	5.7	.004	5.2	3.8	.01	5.2	.16	NS
RIn	8.1	1.1	NS	8.1	.11	NS	8.1	3.3	.003	8.1	.81	NS
Total	73.0	.04	NS	73.0	4.2	.01	73.0	4.6	.003	73.0	.79	NS

Variables mentioned in the first, second and fourth columns have df 2 and the third column have df 3.

*Non Significant

Grand total mean score of ORSS for professors, associate professors, assistant professors, and instructors were 48, 74, 78, and 71 respectively. The stress levels within faculty's academic ranks were compared using ANOVA (table 2). Duncan's post hoc showed that mean scores of S/RC, RI, PI, IRD, RS, RIn, RA, and grand total in professors were significantly different from those in associate, assistant professors and instructors. Professors to some extent showed the lowest scores on almost all dimensions of role conflicts in comparison to the other groups. With regard to employment status, ANOVA results (table 2) and Duncan's post hoc showed that there were statistically significant differences between permanent and probationary faculty with IRD in which the permanent faculty had lower level of role conflict than probationary faculty.

Pearson's correlation test was estimated between various role stress dimensions and experience and age. The results (Table 3) reveal that there are weak but significant and negative relationships between IRD, RS, RE, RA, RO, S/RC, RA, RIn, and grand total with age and experience.

**Table 3:** Pearson's product-moment correlation values.

	IRD	RS	RE	RO	S/RC	RA	RIn	Total
Experience	-.203(**)	-.176(**)	-146(**)	-.102(*)	-.130(*)	-.181(**)	-.212(**)	-.178(**)
Age	-.244(**)	-.155(**)	-.128(**)	-.112(*)	-.130(*)	-.172(**)	-.209(**)	-.176(**)

** Correlation is significant at the 0.01 level (2-tailed).

* Correlation is significant at the 0.05 level (2-tailed).

The results obtained from the multiple linear regression, as shown in table 4, revealed that belonging to basic sciences departments and experience have the strongest contribution to total role stress score. Table 4 shows the results of multiple linear regression analyses, examining the contribution of age, experience, academic rank, employment status and department of affiliation in the ten dimensions of the role stress.

**Table 4:** The results of Multiple Linear Regression Analysis

Dependent Variable	Significant predictors*	β**
Total	Experience	-.177
ORSS score	Dept. of Basic Science	-.160
IRD	Age	-.253
	Dept. of Basic Science	-.224
RS	Dept. of Basic Science	-.171
	Experience	-.126
	Academic Rank	-.136
REC	Permanent status	+.175
	Dept. of Basic Science	-.197
	Age	-.125
	Sex	-3.187
RE	Experience	-.146
RO	Dept. of Basic Science	-3.604
	Age	-2.817
	Temporary status	-2.322
Ris	Dept. of Basic Science	-.109
PI	Dept. of Basic Science	-.149
	Academic rank	-.149
S/RC	Permanent status	+.207
	Dept. of Basic Science	-.186
	Sex	-.107
	Age	-.269
RA	Permanent status	+.172
	Dept. of Basic Science	-.188
	Experience	-.282
Rin	Experience	-.212

* All predictors mentioned in the table have significant F (p < .05). Not significant results were omitted

**Beta is the standardized regression coefficient.

## Discussion

The mean scores in this study show that role stress was experienced comparatively in high amount among faculty members in all three Iranian medical schools, compared to the reported norms [14]. Mean scores of RO, REC, IRD, RIn, RS, and RIs have been found relatively higher than RA, PI, S/RC, and RE dimensions of role stress, but all dimensions of stress were perceived nevertheless by faculty members.

Role overload describes situations in which employees feel that there are too many responsibilities or activities expected of them in respect to their available time, and abilities. [14,23,32]. This was the case in this study, where high level of role overload were found among faculty members.

Role-expectation conflict was the second major role related problem among faculty members. This means they do experience conflicting demands from their colleagues and superiors [15]. The high level of inter-role distance found among participants may show that faculty members feel stressed due to the many roles they perform, which may lead to strain. For example a faculty member may face a conflict between his/her academic role as a teacher/clinician/researcher and social role. These sorts of inter-role conflicts are quite common in modern societies where individuals occupy multiple roles within and out of their organizations [14,15,21].

Resource inadequacy was also found to be at a significantly high level among faculty members. They seem to be more efficient in their organization if they have the autonomy to select tasks and have adequate facilities available to do their job as well as having opportunities to learn new skills [15]. A high level of role stagnation among faculty members was also found. This demonstrates that they feel stress due to covering similar professional roles and dealing with similar type of challenges in medical schools [15,19,33]. Role isolation is the feeling that certain roles are psychologically nearer to a person than others. The gap between desired and existing linkage will indicate the amount of distance between two roles [15]. This was the case in this study for medical faculty members. It may be because of internal politics among other role occupants at the same or a higher level.

Personal inadequacy was also found in relatively high level. This form of role stress takes place when individuals feel they are not adequately skillful, competent and trained to meet the demands of their role. Feeling of personal inadequacy is quite unusual among professors compared to other ranks [15].

Inter-role distance is a conflict between self concept and expectations and the role as perceived by the role occupants. In the present study, inter-role distance was found at a high level. It could be argued that if faculty had the autonomy to take decision for different activities and tasks, they would be doing something different than what they currently do.

The ANOVA analyses showed no significant differences in the mean ORSS scores among the three medical schools, which implies that despite of their differences in size and educational programs, role stress is felt at equal levels. However, the findings of the present study may suggest that medical school faculty members in Iran face role stress possibly due to new levels of complexity and diversity in their roles and responsibilities. In other words, sources of stress are possibly due to working in a broader and more complex clinical field, more responsibilities for a low "reward", a bureaucratic system with insufficient autonomy, and dealing with the many challenges of the process of reform in medical education.

These finding are in agreement with the study on measuring academic production conducted in order to measure faculty activities [31]. The authors state that the combination of rapid technological change and the integration of new business structures into the academic healthcare systems have propelled faculty roles and responsibilities to new levels of complexity and diversity. Therefore, faculty at some medical schools, have increased their clinical activities in response to financial pressures, believing that compensation for research and other scholar activities may be reduced, while teaching activity may be under resourced and inadequately rewarding.

The ANOVA analyses show significant differences in the mean ORSS scores of grand total and dimensions of IRD, RS, REC, RO, SRC, and RA among three groups of departments. It is obvious that according to the variety of departments, the roles of faculty are different. For example, in basic sciences departments, the major roles of faculty would be teaching and research, while faculty members in clinical departments have to undertake clinical work and community healthcare services as well. The results show that the departments of medical basic sciences have lower level of stress and conflicts in comparison with departments of medical clinical and surgical. Therefore, the higher range of role stress among faculty in clinical departments may be attributed to their expanded role of community services in the integrated universities of medical sciences and health services in Iran.

Referring to the relationship between role stress dimensions and academic ranks, it is quite understandable that professors have low scores in all dimensions compared to the other ranks. Instructors, assistant and associate professors had more inter-role distance problem as compared to professors, as they have less experience on their organizational role. Meanwhile, instructors have a different role stagnation compared to professors. It is quite clear that as professors have already reached the apex of their academic career, they don't have stagnation problems compared to instructors and other ranks that still have room for career advancement. Such an argument could be given for other significant dimensions of role conflict related to the academic rank of faculty members [15,19,34].

ANOVA results show that there are statistically significant differences between permanent and probationary faculty with IRD in which probationary faculty had higher level of role conflict. We argue that probationary faculty are less qualified in their multiple roles and about the burden of their organizational role, while permanent faculty are well aware of organization's demands and therefore get properly adjusted.

A number of stepwise regression analyses were conducted to determine the best predictors of role stress. The results obtained show that experience and affiliation to medical basic sciences department are common best predictors of each dimensions of role stress, meaning that longer experience and affiliation to basic sciences decreases the level of role conflict. In view of that, the findings confirm that the department of affiliation, academic rank, length of services, and employment status of faculty members were significant predictors of role stress and role conflict among medical school faculty members [19].

Two limitations of the study should be noted. First this study is focused on medical school faculty members and it has not addressed and compared other non-medical faculty members' role stress perception. Second this study had good sample size with acceptable response rate and also an instrument with confirmed validity and reliability. However, the method of sampling was convenient among medical schools according to their national rank and good access to samples.

## Conclusion

The findings of this study can assist administrators and policy makers to provide an attractive working climate in order to decrease side effects and consequences of role stress and increase productivity of faculty members. Furthermore, it also may help to explore faculty perceptions towards their roles. By better understanding their current situation may help faculty members to develop coping strategies recognized to reduce role related stress. The absence of an effective coping strategy may lead to burnout as a result of role stress. Ultimately, burnout may affect the functioning, and effectiveness of the organization and its employees. Therefore, there should be more social and organizational supports as well as better resources and opportunity to extend collaborative relationship among faculty at different levels and departments, and to develop appropriate coping strategies and eventually other forms of possible interventions considered suitable [16,35].

Our future investigations will look at the effects and consequences of role stress on individuals and organizations, and will suggest interventional measures for decreasing the level of stress among medical faculty.

## List of abbreviations used

ORSS: Organizational Role Stress Scale

IRD: Inter-Role Distance

S/RC: Self/Role Conflict

REC: Role-Expectation Conflict

PI: Personal Inadequacy

RS: Role Stagnation

RA: Role Ambiguity

RO: Role Overload

RE: Role Erosion

RIn: Resource Inadequacy

RIs: Role Isolation

EDC: Educational Development Centers

## Competing interests

The author(s) declare that they have no competing interests.

## Authors' contributions

SA designed the study, abstracted data, performed statistical analysis, interpreted the results, and drafted the manuscript.

TC participated in initial study design, participated in study implementation, participated in statistical analysis and revised the manuscript critically.

IM interpreted the results and reviewed, edited, and revised the manuscript critically.

MB supervised the whole project, participated in initial study design, and revised the manuscript critically.

All authors read and approved the final manuscript.

## Pre-publication history

The pre-publication history for this paper can be accessed here:


